# Editorial Chit Chat

**Published:** 1870-01

**Authors:** 


### EDITORIAL CHIT CHAT.

**Try It.**

A very curious result may be produced in stereoscopic views, by coloring an object that it is desirable to have white, say that of a cat or poodle-dog, on the right side, red, on the left, green. The result will be white.

**Comfortable.**

Those short, plaid, walking dresses worn by ladies this winter. They are cut so as to fit the form loosely, and the waist is lined with heavy flannel. The most sensible dress we have seen for many a day.

**An Offer.**

We have a tin pan which we will donate to the city for a skating pond, provided they will construct a suitable rink-building to contain it. The pan is about the size of most rinks.

**Strong Team.**

J. A. TOWNER, Esq., the popular writer, and R. M. WATTS, Esq., the most skillful job printer anywhere in these parts, have been engaged by the new *Advertiser*, which is to make its appearance on the 1st of January.

**An Apology.**

We have been quite ill for the past two months which fact must account for the lack of editorial matter in this number of the BISTOURY. We hope to be able to complete several articles upon matters of health by our next issue.

**That Orchestra.**

Mr. Isaac Green has organized a new orchestra with new music, and is treating the patrons of the Lecture Course, at the Opera House, with as fine string music as one could wish for. It is now really a pleasure to go early to the lectures and listen to Mr. Green's orchestra.

---

**Dear Goose.**

We have an acquaintance in this city who, in company with three others, traveled five hundred miles to capture one wild goose. They succeeded—as they deserved to, at an expense of $250 00, and brought the goose to this city "Wild goose chases" are said to be generally unprofitable—this is no exception to the rule. His gooseship may be seen alive and healthy, at the residence of one of the nimrods of this city.

---

**Appreciated.**

We are glad to see the BISTOURY taken so extensively by the profession. About one half of the orders we now receive come from them. Their letters always contain kind words of encouragement and praise, for which we are very grateful. We will endeavour to make the BISTOURY as useful to the profession during the coming volume as it has been in the past. We hope thus to merit and retain their patronage.

---

**To the Point.**

On the tenth anniversary of our wedding we received a package, from some joker in Philadelphia, containing a huge instrument, manufactured from tin, and in the shape of a BISTOURY, bearing the following inscription:—"*The sharpest of Medical Journals.*" Now, that's what we call a sharp joke—it's to the point too. We have it suspended in our sanctum where it can be used in severing the hymenial tie of any who stand in need of such an operation. As for us, we have not yet applied for a divorce, and therefore do not desire to put it to the test at present.

---

**A Fine Bust.**

Conkey has completed the bust of John M'Gee, Esq., in marble. It is an excellent likeness and a splendid piece of modeling. Mr. Conkey has added greatly to his fame as an artist, in his production of this bust. Now that the County Commissioners have purchased the vacant lots next the Conrt House, we hope they will give Mr. Conkey a commission to erect a suitable Soldier's Monument upon the spot. It is a duty we owe our soldier dead, and would be a handsome complement to a skillful and deserving home artist.

**New Cards.**

Our readers will notice several new cards on the cover of this journal, among which is that of Mr. Jaques, of Waverly, N. Y., who takes advantage of the large circulation of the Bistoury in Waverley, Factoryville, Athens, Sheshequin, and Towanda to tell the people of those places, where to buy hardware and stoves. His store is a large one and well stocked with everything pertaining to the hardware trade. Everybody in need of such ware will call upon Mr. Jaques of course, as he is a liberal advertiser and can therefore afford to sell goods much cheaper than his neighbors.

---

**"'Tis Sweet to be Remembered."**

So says an old song, and if the sentiment is a correct one, as we doubt not it is, we must rival rock-candy in saccharine development. Recently we were reminded by our friends both at home and abroad, that the tenth anniversary of our wedding day had arrived; and such a flood of tin ware that poured in upon us from every direction, was marvelous to behold. We have taken an inventory of our stock in hand and find it to consist of the following articles, useful and otherwise, particularly the latter, viz.—One fine plate oven; bread chest; 3 coffee chests; farina kettle; potato steamer; pudding mould; 4 tea kettles; 1 dozen muffin rings; 1 dozen patty pans; 5 pails of various patterns; spice box; 3 coffee pot stands; 3 jelly moulds; 2 fruit baskets; egg-beater; scoop-shovel; a bistoury, 6 ft. long; 3 tin hats, all No. 7 1-8 and in the *height* of fashion; 1 cap; a full set of jewelry, watch and chain, ear-rings, breast-pin, bracelets, and finger-ring; 1 large bread pan; a dish pan large enough to take a plunge bath in; patent mouse trap; young man's companion; a trotting horse—time two min.; brook trout, caught on "Great Dun," in forty fathoms of water; card of regrets, 15 by 24 in.; 1 latest style bonnet, with feathers, flowers, and ribbons complete; a family grater; "shoo fly," in case; 6 horns, of various sizes, shapes, and tones; 2 canes; pair spectacles; 2 swords; 2 boxes seidletz powders; candle sticks and moulds; 2 funnels; 3 rattle boxes; match safe, a speculum and double action syringe; an elegant boquet in tin holder; tin cups and tin plates, &c., &c., *ad infinitum.*

We intend adding the collection to our cabinet of specimens in natural history, after which it can be leased for exhibitions at church fairs, &c.

**The Next Volume.**

We expect to make several improvements in next issue. It will be better printed and on better paper. Hope our subscribers will be prompt in sending in their subscriptions for the next year. All moneys received to date are credited on the present volume *only*, for which the back numbers have been sent. Please be prompt in your remittances and we will give you a real useful, wide-a-wake journal for a very small subscription price. Payments are now due. Send the money promptly and you will greatly aid us in our work.

**Excavations at Jerusalem.**

LIEUT. WARRAN, of the British navy, has just made an additional report upon his excavations at Jerusalem. He says that he has discovered large foundation stones seventy feet under ground, which seem to have been dressed at the quarries with seven-pointed chisels, for after diligent search *he could find no remnants of tools.* Some of the stones have three letters cut on them, painted blue and red apparently, which Lieutenant Warren takes to be masonic marks, but which he prefers to have examined in the British Museum, and he has sent several of the stones to London for that purpose. He thinks he is gradually working towards the foundation stone of the Temple, in which he expects to find extraordinary Masonic emblems, jewels, relics, and writings.

**1870.**

Look out! Scientific men have discovered that the sun is shooting out from itself, streams of what they call magnetic light, which possess great power and heat. It is approaching the earth's orbit at the rate of about three and a half million miles a month. It has already accomplished half the distance. Should this be a fact, conjecture will not long have to exercise itself to discover its effect upon our globe. Since 1866, wise men and religious men have been looking out for a fulfillment of some of the last of the Biblical prophecies. This look-out is no novelty but the reasonableness of it seems to be better sustained now by the sign of time than ever before. We cannot tell, no one can tell, but all can obey our preliminary injunction—Look Out?

**Parisian Houses.**

The idea of building houses in crowded cities with the kitchen in the attic, prevails largely in Paris and has very many good reasons for general adoption. Though food is the foundation of our existence there is no necessity that the cooking of it should be done in the foundation of our houses. The odors that arise from the kitchen should not pass through parlor and bedroom before reaching the high heavens, as they do in our cities when room is built over room, to the height of three or four stories. Coal and raw materiels could be elevated to the attic about as inexpensively as they can be lowered to the basement. The dry-room of the Laundry could be on the roof, and servants would be much more under the control of masters and mistresses. There would be less running out and in, and no gossiping over the area railings. The many advantages this style of house posesses commends it to the attention of builders.

**Cardiff Giant.**

The best explanation of this notorious sensation to our mind, is that the gypsum rock from which it was cut is ancient but that the shaping of it to its present appearance is recent. That is, that some one familiar with the cutting of marble, saw the rock, and its shape suggested the experiment which has resulted so admirably in a money point of view. The apparent antiquiety of the thing is real, but existed before it possessed its present shape. Thus, we in our younger days took once from a pine board, a knot that Nature had curiously formed into something of a likeness of a man's head. With a very little scraping and cutting, the knot became a likeness of a friend of ours. We did not try to pass it off as an object of antiquity, but suceeded in making our friend believe that we had hewed it from the rough, and obtained many compliments for our skill. It was our only attempt at "sculpin," lest we should lose our reputation.

Is it not more than probable that the "Cardiff" is susceptible of a similiar explanation?

**Measurements and Weights.**

A well-proportioned man

| | | | | | | |
|---|---|---|---|---|---|---|
| Measuring | 6 feet | | | should weigh | 175 | pounds. |
| " | 5 | " | 11 inches, | | 170 | " |
| " | 5 | " | 10 " | | 165 | " |
| " | 5 | " | 9 " | | 160 | " |
| " | 5 | " | 8 " | | 155 | " |
| " | 5 | " | 7 " | | 150 | " |
| " | 5 | " | 6 " | | 145 | " |
| " | 5 | " | 5 " | | 140 | " |

and so on, for every inch in height above five up to six feet, an additional five pounds should be added to the weight. This table has been estimated from measurements and weights of 2,650 persons.

